# Adherence to the Gluten-Free Diet Role as a Mediating and Moderating of the Relationship between Food Insecurity and Health-Related Quality of Life in Adults with Celiac Disease: Cross-Sectional Study

**DOI:** 10.3390/nu16142229

**Published:** 2024-07-11

**Authors:** Nour Amin Elsahoryi, Mohammed Omar Ibrahim, Omar Amin Alhaj

**Affiliations:** 1 Department of Nutrition, Faculty of Pharmacy and Medical Sciences, University of Petra, Amman 11196, Jordan; omar.alhaj@uop.edu.jo; 2 Department of Nutrition and Food Technology, Faculty of Agriculture, Mutah University, Karak 61710, Jordan; mohammedomar@mutah.edu.jo

**Keywords:** celiac disease, food insecurity, health-related quality of life

## Abstract

This cross-sectional study aimed to estimate the relationship between food insecurity (FI) and health-related quality of life (HRQoL) in patients with celiac disease (CD) and assess whether this relationship is mediated or moderated by adherence to the gluten-free diet (GFD). The results of 1162 samples of patients diagnosed with CD showed that 8.3% of individuals who have a combined score of less than 13 exhibited excellent or very good adherence to the GFD. Furthermore, moderate and fair to poor adherence to the GFD was demonstrated, respectively and 71.9% of the patients had poor HRQoL levels. A significant and moderate positive correlation between FI and GFD adherence (r = 0.489) was found, indicating that 24% of the variance in FI is shared with GFD adherence. The patients’ gender, marital status, and monthly income made statistically significant contributions to the prediction (*p* < 0.05). Females had significantly lower HRQoL scores than males (B = −4.619, 95% CL: −6.08, −3.16) after holding all other variables constant. In conclusion, FI is mediated by GFD adherence to HRQoL. Moreover, a significant total effect relation was found between HRQoL and both FI and GFD adherence, suggesting that lowering symptoms and complications associated with CD may reduce the impact of FI on HRQoL.

## 1. Introduction

Celiac disease (CD) is an inherited autoimmune disorder caused by the ingestion of the gluten allergen, a protein found in wheat, barley, and rye, leading to damage to the small intestine and, consequently, nutrient malabsorption [1,2]. The incidence of CD has increased worldwide over the last few decades; 7.5% is the average annual increase among different populations [3]. This is attributed to the high amounts of gluten fed to newborns after breastfeeding was stopped [4] or may be due to the use of antibiotics during the first year of life, which may increase the chance of developing CD, including a dose-gradient impact [5]. Moreover, it is related to fewer microbes’ exposure during early life, which leads to an overactive immune response in later life [6]. As chronic allergy disease [2,7], Until now, the only currently available treatment is a lifelong commitment to a gluten-free diet (GFD) and strict abstention from foods that contain or are contaminated with gluten protein [1,2]. Patients with CD find this matter uneasy, challenging, and restrictive for several social and financial reasons [8]. On the other hand, gluten-free foods that are not contaminated with gluten and are considered safe for patients are expensive and have limited availability in markets in various countries [9,10,11,12,13], including Jordan. In this way, patients who suffer from a lack of available health resources as a staple food (in other words, they have food insecurity (FI)) have difficulty adhering to a healthy life and therefore unstable health [9,11,12,14]. Many studies indicate a decrease in the health-related quality of life (HRQoL) in patients suffering from CD for several reasons [9,12]. The most important of which is their poor health condition, which is linked to their lack of adherence to a suitable diet, which may be caused by the lack of the main standards that represent food security for them, such as the presence of safe food or economic issues to have healthy GFD [11,12].

On the other hand, strict adherence to GFD diminishes the risk of gluten exposure, which can prompt symptoms and lead to long-term damage to the small bowel [8,15]. Furthermore, adhering to the GFD helps decrease long-term consequences such as damage to the mucous membrane, inflammation, and impaired absorption of essential nutrients like calcium, vitamin D, iron, vitamin B12, folic acid, and zinc [8]. These deficiencies can result in severe conditions like osteoporosis, anemia, and delayed growth [8]. In addition, longitudinal studies have demonstrated that the dietary management of CD reduces the disease burden and improves (HRQoL), besides inducing a histological recovery [16,17,18].

According to Patrick and Erickson (1993), the definition of HRQoL is “the value assigned by individuals or society to the duration of life modified by impairments functional states perceptions and social opportunities brought about because of disease injury treatment or policy” [19]. HRQoL has gained increasing attention in medical research and clinical practice. It provides a comprehensive method for evaluating the effects of medical interventions. Patients’ attitudes are greatly affected by their dietary commitment. This emphasizes the importance of understanding their self-perceptions. This is especially true for chronic diseases that require lifelong lifestyle changes [20]. The ideal adherence to the GFD is important, but the task is demanding; the patient faces a learning process, the necessity of finding a new motivation, and adjusting to the new behavior [17,18]. FI is a significant barrier and a prevalent and important public health concern for patients with CD [21]. It includes the availability of gluten-free food and food options for a suitable diet [21,22,23]. FI is defined as a lack of food accessibility to meet safe, healthy, and nutrient needs. Diet quality and variety fall short of a person’s requirement and can be irregular, resulting from the need to replace healthier food with fewer, low-quality options [21,24]. For patients with CD, FI indicates the availability of gluten-free products with lower gluten content than the cutoff [25].

FI has been linked to limited availability of food, reduced variety or quality of the diet, and inadequate quality of food. Reducing food intake, reducing variety, and stress or financial problems can all impact the way we approach FI and GFD adherence [21,22,26]. To the best of our knowledge, the role of FI in the lives of adults with CD and HRQoL has not been explored. Therefore, understanding the HRQoL, the role of FI among patients with CD, and the relationship between these variables and GFD adherence is needed. This population-based study will help fill the gap in the current literature; as such, a study has not been conducted on FI in people with CD and HRQoL. This study also aims to further elucidate the complex interplay between FI, HRQoL, and GFD adherence in vulnerable adult populations whose disease has been characterized by GFD. The benefit of GFD lies in the reduction of unsuspected gluten exposure, the resolution of duodenal pathology, the improvement of HRQoL, and the reduction of CD-related comorbidity [27]. Nonetheless, successful long-term adherence to the GFD is challenging, especially when FI is present. This is a significant barrier to CD therapy and a public health issue. FI leads to non-adherence to the GFD, thus leading to non-improvement in HRQoL, which is in part due to challenges with finding safe gluten-free options as well as access to nutritious alternatives.

A conceptual model was employed in this study to examine the effect of moderation and mediation [28]. A moderator variable in a moderation model has the ability to change the direction of the association between a predictor and an outcome variable, as well as to increase or decrease it. On the other hand, in mediation models, a mediator variable provides an explanation for the relationship between the predictor and outcome variables.

This investigation designated adhering to a GFD as the moderating/mediating variable. The moderation model postulated that adhering to a GFD would interact with HRQoL, influencing the association between HRQoL and FI. Meanwhile, the mediation model proposed that individual levels of good adherence to a GFD would facilitate the relationship between HRQoL and FI. The conceptual framework relied on a mediation model, hypothesizing that GFD adherence may explain, at least partially, the impact of FI on HRQoL. The moderation model investigated whether the relationship between FI and HRQoL varied depending on the level of adherence to the GFD. FI was found to have important implications for the HRQoL; these are impacted by several socio-economic factors, including gender, marital status, and income.

Research has shown that food insecurity can affect males and females differently; females, particularly when they are pregnant or have children, may experience higher levels of stress and compromised dietary intake when facing FI [29,30]. Moreover, female patients with CD may have more barriers to accessing and affording a GFD, which is essential for managing their condition. This could lead to poorer health outcomes and a lower quality of life compared to male patients with CD [29]. Furthermore, married individuals may have better access to financial resources and social support, which can buffer against food insecurity. Conversely, single, divorced, or widowed individuals may face greater challenges in securing adequate food, which can exacerbate the management of CD and reduce life quality [31]. In addition, income could play a crucial role in FI, whereas higher income levels can correlate with better access to nutritious foods, including GFD [32].

On the other hand, lower-income individuals may struggle to afford specialized GF products or have limited access to grocery stores with a wide range of GF products [33]. Therefore, interventions aimed at improving FI among patients with CD should consider these factors to effectively support HRQoL. The study intended to provide valuable insights into these relationships in the specific context of patients with CD managing their condition through dietary restrictions. We hypothesized that (a) adherence to GFD moderates the relationship between FI HRQoL among the patients with CD. In other words, the direction or strength of the correlation between FI and HRQoL changes depending on how strictly the GFD is followed (Moderation Hypothesis), and (b) adherence to the GFD is a factor mediating the relationship between FI and HRQoL. In other words, adherence to a GFD partially explains the impact of FI on HRQoL among patients with CD (Mediation Hypothesis).

The importance of this study confirmed the harmful consequences and complications of poor adherence to GFD, which may progress to poor symptom control and an increase in the risk of gastrointestinal cancer [25,31].

Therefore, this study’s foremost objective is to estimate the relationship between FI and HRQoL in patients with CD and assess whether this relationship is mediated or moderated by adherence to the GFD. Guided by the conceptual models, we formulated two hypotheses: (1) adherence to the GFD serves as a moderator, and (2) adherence to the GFD serves as a mediator of FI and HRQoL among adults with CD. The evidence of (1) and/or (2) would indicate that adherence to the GFD functions as a supportive factor of the HRQoL.

## 2. Methods

### 2.1. Study Design and Participants

This cross-sectional study was conducted between January and June 2023, focusing on adults with CD residing in Jordan. A convenience sample (*n* = 1162) of individuals with CD was selected from Jordan’s “Celiac Care Providers Society” membership list. A web page survey was sent using the WhatsApp application (version number: 2.19.134). by the Association to those who met the eligibility criteria. The principal goal of this study was introduced to respondents in a message on an announcement board. All subjects gave their informed consent to participate in the research by filling in the initial page of the questionnaire. In order to become a study participant, all participants had to be between the ages of 18 and 65 years and have some kind of diagnosed CD, according to some self-reported methods (biopsy or blood test and biopsy). Importantly, this diagnostic approach approximated that employed in another UK-based survey [9]. Participants with other conditions that potentially influence their (HRQoL), such as multiple food allergies, diabetes mellitus, hepatitis C, inflammatory bowel diseases, kidney transplantation, and multiple sclerosis, were excluded from participation.

### 2.2. Sample Size

A sample size calculation revealed that a minimum of 385 patients with CD would be necessary to achieve a 95% statistical power and a two-sided percentage significance level of 5% (i.e., a *p*-value less than 0.05 in both directions). This calculation was based on the sample size equation tailored for cross-sectional surveys, primarily due to the absence of recent, precise prevalence data on CD among adults in Jordan [34,35]. The sample size was determined using the Raosoft online calculator (Raosoft, Seattle, WA, USA), (©Copyright 1999) a specialized tool for population surveys that considers factors such as the desired confidence level, margin of error, and population size [36,37].

### 2.3. Data Collection

Data were collected using a webpage (electronic and self-reported) questionnaire completed once by each participant. This questionnaire is divided into four parts. The first part included the demographic characteristics of the participants, such as age, sex, marital status, monthly earnings, level of education, and body mass index (BMI). The second part was an HRQoL questionnaire [38], while GFD adherence [39] was the third part; the last part covered the Household Food and Access Insecurity Scale (HFAIS) [40].

### 2.4. Adherence to a GFD

Adherence to the GFD-validated questionnaire (Cronbach’s α = 0.809) was used in this study [39]. The questionnaire included seven questions designed to evaluate several aspects of the diseases, such as symptoms experienced by patients with CD, knowledge about how they handled themselves, reasons as to why they adhere to a GFD, and how much they stick to this type of feeding program. Each question was presented on a five-point Likert scale ranging from “None of the time” to “All of the time”. The scores on this survey were between 7 and 35, and higher scores showed poor adherence towards GFD. They are Excellent or Good (less than 13), Moderate (13–17), and Poor (>17). The English questionnaire was translated into Arabic to facilitate effective communication between the data collectors and the respondents. This marked the first time this questionnaire was translated into Arabic for utilization in Jordan.

### 2.5. Psychometric Analysis of the Adherence to a GFD Questionnaire

To generate the Arabic questionnaire, we employed the forward-backward translation method, as detailed by [41]. Initially, an Arabic-English bilingual researcher translated the survey from English to Arabic. Subsequently, a second bilingual researcher retranslated the questionnaire from Arabic back to English. The two English translations (the original and the translated-back version) were then compared to ensure the accuracy of the item meanings.

To enhance the psychometric validity of the questionnaire, a pilot sample of 11 individuals with CD was employed to evaluate the Arabic version. This pilot study took place in January 2023. Ten working days after the initial distribution of the questionnaire, participants were contacted and received the questionnaire once more via the WhatsApp messaging application. Construct validity was assessed by calculating the correlation between the items on the scale using correlation matrices. Reliability was evaluated through internal consistency (Cronbach’s alpha), and test–retest reliability (Pearson’s correlation coefficient) was used to calculate the test–retest reliability. For the participant’s privacy, unique identifier codes were assigned to the questionnaires. The questionnaire was reliable regarding the overall internal consistency (Cronbach’s alpha = 0.82) and test–retest reliability (Pearson (r^2^) = 0.94). The alpha coefficient result reflects excellent internal consistency. The results of the stability coefficient indicated strong test–retest reliability, suggesting that measurement error of the questionnaire was less likely to be attributable to changes in the questionnaire.

### 2.6. Household Food and Access Insecurity Scale (HFAIS)

The household FI status is using the Arabic version of the FI Experience Scale Survey Module (FIES-SM) [40]. It is worth noting that the FIES-SM has been rigorously validated for application in Middle Eastern countries with 0.91 for Cronbach’s α [17,40]. This scale comprises nine questions about food consumption patterns over the past 12 months. Respondents answered these questions with a simple “yes” or “no”, denoted as 1 or 0, respectively. The total scores on this scale can range from 0 to 9, depending on the number of affirmative responses. Higher scores denoted elevated levels of FI. Households were classified into four tiers of FI, including food secure, mildly FI, moderately FI, and severely FI, based on the extent of affirmative responses to statements concerning increasingly severe conditions and/or experiences [17,40]. The questions assessed the FS regarding the GFD. For example, question one assessed the availability of gluten-free processed foods, such as gluten-free bread, pasta, breakfast cereal, flour, and snacks, within grocery stores in Jordan. These challenges encompassed factors such as high pricing, proximity to grocery stores, physical disabilities, unavailability, limited variety of gluten-free items, low quality of GFD products, and the absence of gluten-free options in the Jordanian market.

### 2.7. Health-Related Quality of Life (HRQoL)

HRQoL was evaluated using the valid Arabic version (0.84 for Cronbach’s α) of the short-form health survey (SF-12) [38]. The questionnaire consists of 12 items that assess eight dimensions: physical function, physical role, body pain, general health, vitality, social function, emotional role, and mental health. Typically, these dimensions are grouped into two components known as physical health (PCS) and mental health (MCS) [38]. The assessment comprises 35 potential response choice indicator variables. For instance, the question related to physical functioning offers three response choices: 1 (yes, limited a lot), 2 (yes, limited a little), and 3 (no, not limited at all). PCS and MCS are calculated based on the responses to these 12 items [42]. Individuals scoring 50% and above on the total mean were categorized as having good HRQoL, while those scoring below 50% were classified as having poor HRQoL [43].

### 2.8. Ethical Approval

The Research Ethics Committee at the University of Petra in Amman, Jordan, granted ethical approval for this study following the Declaration of Helsinki (Grant number: Q1/1/2023). It is important to note that participation in this study was entirely voluntary, and each participant had the right to withdraw at any time.

### 2.9. Statistical Analysis

Data were analyzed using the Statistical Package for the Social Sciences (SPSS) version 25, including mean and standard deviation for continuous data and frequencies and percentages for categorical variables. The monthly income was divided into three dummy coded variables: JOD 500–700, more than JOD 700, and less than JOD 500, with less than JOD 500 as the reference group. Personal correlation was used to determine the link between the primary study variables (FI, GFD adherence, and HRQoL). Multiple linear regression analysis was used to find the predictors of HRQoL.

Following Hayes’s (2022) guidelines [28], the SPSS PROCESS macro was used to test the hypotheses on the effects of moderation and mediation. The mediating effect of GFD adherence was tested in five steps (H2)—(1) direct effect of mediator (GFD adherence) on HRQoL, (2) direct effect of predictor (FI) on mediator (GFD adherence), (3) total effect of predictor (FI) on HRQoL, (4) direct effect of predictor (FI) on HRQoL with inclusion of mediator (GFD adherence), and (4) using SPSS PROCESS macro, a 1000-sample bootstrap procedure was used to estimate bias-corrected 95% confidence intervals (CIs) to test the significance of the indirect effect of the relationships (*p*-value ≤ 0.05). If the CLs did not contain 0, indirect relationships were seen as significant, indicating a significant mediating effect (Hayes, 2022 [28]).

Full mediation is presented when the beta weight is reduced, and the *p*-value is not significant, while partial mediation is presented when the beta weight is reduced but the *p*-value is significant (Hayes, 2022 [28]). Significant predictors of HRQoL were entered as covariates in the moderation and mediation models.

To test for the moderation effect (H1), the relationships for (1) the direct effect of predictor (FI) on HRQoL, (2) the direct effect of a moderator (GFD adherence) on HRQoL, and (3) the direct interactions effect (FI × GFD adherence) on HRQoL had to be significant. In SPSS PROCESS, the interaction effect is calculated automatically via the software, and it also produces the proportion of the variance explained by the moderating effect of GFD adherence (R square increase due to interaction).

## 3. Results

### 3.1. Participants Characteristics

This cross-sectional study comprised a convenient sample of 1162 individuals diagnosed with CD: 80.5% had been diagnosed through biopsy, and 19.5% were diagnosed by a combination of a blood test and biopsy, with females being predominant, as shown in Table 1. The majority were unmarried, and their educational background varied. Most of the participants held primary or secondary degrees. Approximately half of the participants reported an income below JOD 500. Taking into account that the average monthly wage in Jordan was JOD 535 in 2000 and increased to JOD 691 in 2021, according to the results of the General Statistics Department. Based on the self-assessment carried out by the patients in this study, the results showed that the majority of them suffer from being overweight, which is described by the BMIS, as shown in Table 1.

### 3.2. Exploring FI Patterns, GFD Adherence, and Correlations with HRQoL among Patients with CD

**Figure 1:**
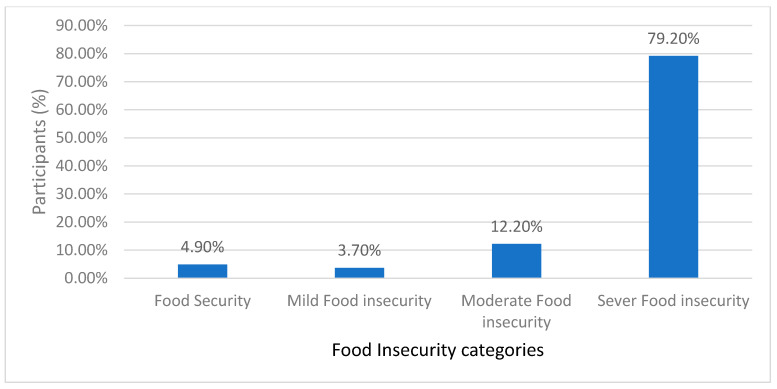
Food Insecurity (FI) level in patients with Celiac Disease (CD).

**Figure 2:**
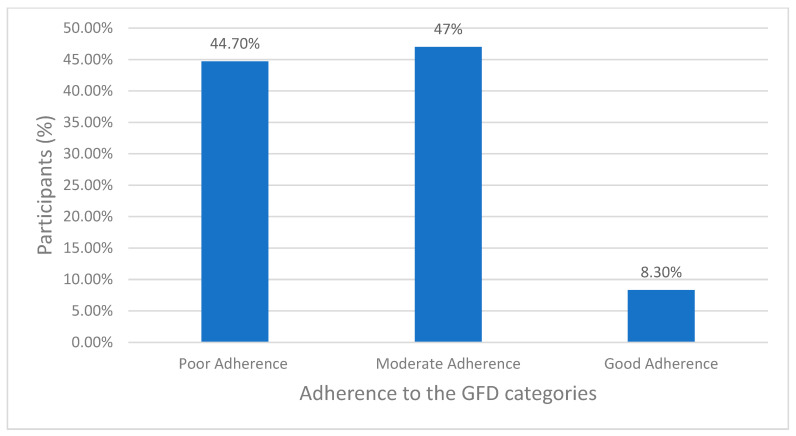
Adherence level to the gluten-free diet (GFD) in patients with Celiac Disease (CD).

**Figure 3:**
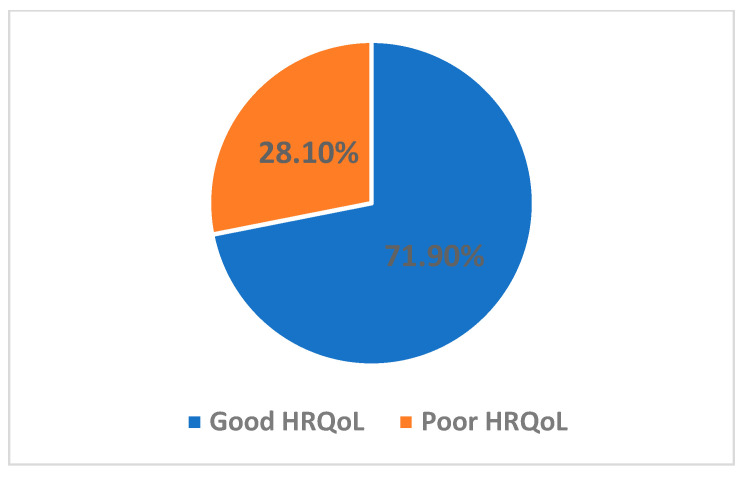
Health-related quality of life (HRQoL) percentage in adults with Celiac Disease (CD).

As shown in Figure 1, the vast majority of patients with CD in Jordan suffered from severe FI, while a very small percentage were food secured. Based on the GFD adherence scoring, a small percentage of the participants who scored less than 13 exhibited excellent or very good adherence to the GFD, as shown in Figure 2, whereas the majority demonstrated moderate and fair-to-poor adherence to the GFD, respectively. Regarding the HRQoL, two-thirds of the patients in this study with CD reported poor HRQoL, as shown in Figure 3. As shown in Table 2, Pearson’s correlations revealed a significant moderate positive correlation between FI and GFD adherence, indicating that 24% of the variance in FI is shared with GFD adherence. Further, Pearson’s correlations revealed significant negative relations between FI and GFD adherence, sharing 13.5% and 12.5% of the variance with HRQoL, respectively, both with medium effects, as shown in Table 2.

### 3.3. Predictors of Health-Related Quality of Life (HRQoL) in Patients with CD: A Comprehensive Regression Analysis

Multiple linear regression was conducted to predict HRQoL, incorporating five variables, including age, BMI, gender, marital status, monthly income, and education levels. The assessment revealed linearity through partial regression plots and studentized residuals plotted against predicted values. A Durbin–Watson statistic of 2.09 confirmed the independence of residuals, while homoscedasticity was observed through visual inspection of studentized residuals versus unstandardized predicted values. The absence of multicollinearity was indicated by tolerance values exceeding 0.1. No studentized deleted residuals exceeded ±3 standard deviations (SD), leverage values were below 0.2, and Cook’s distance values were below 1. The assumption of normality was met, supported by a Q–Q Plot.

As shown in Table 3, the multiple regression model significantly predicted HRQoL. In addition, gender, marital status, and monthly income categories of JOD 500–700 and JOD > 700 made statistically significant contributions to the prediction. By holding all other variables constant, females had significantly lower HRQoL scores than males. Being married decreased the HRQoL score by 2.70 compared to being single without a spouse. Participants with an income between JOD 500–700 or higher than JOD 700 exhibited a significantly positive association with HRQoL, holding other independent variables constant.

### 3.4. Mediation and Moderation in the Context of Food Insecurity’s (FI) Influence on Health-Related Quality of Life (HRQoL)

Process Model 4 [44] analysis was used to explore the roles of GFD adherence as both a mediator and moderator as shown in Table 4.

**Figure 4:**
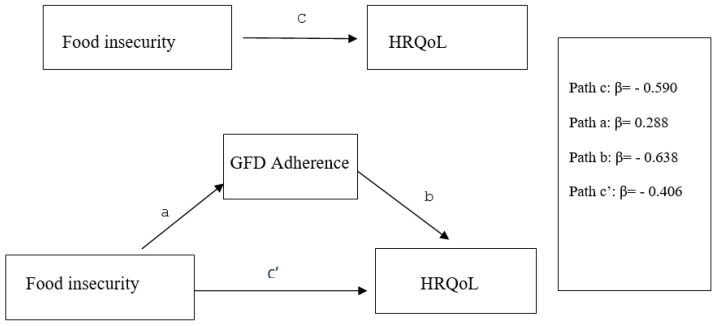
Conceptual framework of the potential mediating effect of adherence to the GFD on the relationship between FI and HRQoL in adults with Celiac Disease (CD). Analysis was adjusted for gender, marital status, and monthly income.

Mediation analyses were conducted on the entire sample of 1162 participants. This model explained 21.3% of the variance in the HRQoL score. The results indicated a significant total effect between HRQoL and both FI and GFD adherence. Upon introducing GFD adherence into the relationship between FI and HRQoL, the direct effect remained significant (*p* < 0.001). This reduction in the direct effect, referred to as partial mediation, was complemented by an indirect effect β coefficient of −0.184, with CI 95% = −0.236 to −0.133 (excluding zero). Therefore, GFD adherence is considered a mediator for FIon HRQoL. Figure 4 shows the output model for the mediation effect of GFD adherence.

Based on the analysis conducted with SPSS’s PROCESS macro, the full model of all variables (gender income, marital status, FI, GFD adherence, and the interaction between FI and GFD adherence to predict perceived stress was statistically significant, as shown in Table 5. The interaction between FI and GFD adherence was not significant *p* = 0.965), indicating that the relationship between FI and HRQoL was not moderated by GFD adherence.

## 4. Discussion

This study aims to estimate the relationship between FI and HRQoL in patients with CD and assess whether this relationship is mediated or moderated by adherence to the GFD.

Due to the many economic barriers to GFD adherence, the aspect of FI has become an essential factor in evaluating the availability of a sufficient diet; the authors of [45,46] reported that not only economic factors play a decisive role in FI but also the social and cultural factors have a significant interpretation. To clarify the picture, Taghdir et al. (2016) documented that education on nutrition and increasing awareness at the community level about the disease can effectively enhance the level of FI among patients with CD [33]. The majority of the current study patients with CD were insured, unable to fulfill their nutritional needs, and had poor adherence to appropriate GFD. These results may be attributed to the low bioavailability of gluten-free products, the limited role of community nutritionists in counseling and education regarding gluten-free products, insufficient scientific awareness of consumers about these products, misleading labeling of some commercial foods and beverages, and the incorrect preparation methods of GFD meals either in-home or in restaurants [47,48,49,50]. To our knowledge, few studies have assessed the effect of FI among patients with CD.

A low percentage of adherence to GFD among patients with CD in our study was obvious. The current results were not in agreement with other previous studies, which reported that most patients with CD in our adhered to a GFD [39,51,52]. However, the explanations of such variation may be explained through previous studies investigating factors that influence dietary adherence. In a study conducted by Muhammad et al. (2019) to identify and improve adherence to GFD in people with CD, they found many difficulties in adherence to a GFD, and there are a wide range of barriers for patients with CD to overcome [8]. 

On the other hand, previous studies reported that socio-demographic factors play an important role in adherence. Halmos et al. (2018) argued that males had better stick to GFD than females [39]. On the other hand, Hall et al. (2013) observed no significant difference between sexes with respect to adhering to a GFD [40]. Concerning age, Olsson et al. (2009) found that young people are concerned about isolation and stigmatization for following a GFD [41]. Conversely, patients who are diagnosed with CD in adulthood reported relatively better adherence to GFD [53,54]. Additionally, dietary counseling and follow-up visits among patients with CD resulted in increased adherence to GFD and improved quality of life [55,56]. Moreover, mental disorders such as depression may have negative effects on adherence to the GFD [7,57]. Other reasons why patients do not adhere to GFD include ignorance about foods that contain gluten, such as wheat flour, and inability to read and understand food packaging language [8,48]; high cost of maintaining a GD diet [49,50]; and limited access to pre-purchased GD meals [51,52].

The concept of HRQoL is a critical issue amongst patients with CD. The results of the current study indicated that a high percentage of our study sample had poor HRQoL. The current results were in accordance with a follow-up study conducted by Deepak et al. (2018) on consecutive patients with suspected CD [6]. They documented a reduced HRQoL in adult patients with CD. In the same context, Zysk et al. (2018) reported that patients with CD experience several problems related to their eating behaviors, physical activity, and lifestyle, which may affect their general HRQoL [58]. Furthermore, other studies have reported that HRQoL is poor among patients with CD [17,59]. 

On the other hand, gender, marital status, and monthly income were among the most important predictors of HRQoL in patients with CD among our study population. Females in our study have shown significantly lower HRQoL scores, and these results were in accordance with previous studies, which documented that CD women experience poorer general well-being than CD men [58]. Economic status plays a key role among patients with CD.

The current results have shown that higher income among patients with CD is associated with higher HRQoL scores. This result could be explained by the results of a study conducted by Pourhoseingholi et al. (2017), which documented that GFD products may be expensive and challenging for patients with CD [60]. Consequently, purchasing such products requires higher income. In the literature, there is almost no information about the influence of marital status on the HRQoL among patients with CD. However, an explanation of our result that being married was associated with poor scores of HRQoL is that higher family members divert the purchasing process to food products other than gluten-free products to meet the family’s needs.

Our study focused on the direct relationship between two main aspects, FI and HRQoL, among patients with CD. This relationship is very important, and the results of our study indicated that there was a significant negative correlation between both aspects. Generally, this finding is logical and expected because the more FI, the lower the degree of quality of life. In a recent study conducted by Aljahdali et al. (2024), they found that FI was positively correlated with multiple adverse health outcomes and poorer quality of life and mental health [53]. Based on what we mentioned previously, our study also focused on the direct relationship between FI and HRQoL by testing the effect of adherence to GFD on the relationship between FI and HRQoL. This factor is crucial among patients with CD, and our study results have shown a significant negative correlation between higher scores of adherence to a GFD and HRQoL. This result clarifies that good adherence to GFD will be associated with good HRQoL outcomes. Our study’s results agree with three previous studies conducted among patients with CD.

The first multicenter study conducted by Casellas et al. (2008) was a prospective, cross-sectional study of patients with CD. They documented that good adherence to GFD results in improved HRQoL [54]. The next study was a prospective follow-up study conducted by Deepak et al. (2018) on consecutive patients with suspected CD [6]. They reported a reduced HRQoL in patients with CD, which significantly improves good adherence to GFD. The third study was a meta-analysis performed by Rustagi et al. (2020) to determine the interaction between adherence to GFD and HRQoL [55]. They investigated the fact that partial adherence to GFD is associated with lower HRQoL when compared to strictly adherent patients with CD and that better adherence to the GFD leads to favorable HRQoL outcomes. From a psychological point of view, the weak accessibility to GFD may increase stress responses like anxiety and depression, which may partially explain the association between poor adherence to a GFD and reduced HRQoL [56]. Furthermore, a recent Jordanian study found that the prevalence of anxiety and depressive symptoms among patients with CD was 85% and 82.7%, respectively [57].

The aforementioned findings emphasize the key role of good adherence to GFD on the outcomes of HRQoL and thus, indirectly, the relationship between FI and HRQoL among patients with CD. Therefore, one of the ultimate goals of our study was to examine the moderation and mediation effects of good adherence to GFD on the relationship between FI and HRQoL. The results of our study revealed a positive correlation between the scores of FI and scores of adherence to GFD. This means that lower scores of FI reflect more security, which is associated with good adherence to GFD. Consequently, the later relationship improves, and good adherence to GFD would help to elucidate the relationship between FI and HRQoL. Using mediation analysis, our study supports the hypothesis that adherence to GFD acts as a full mediator of the association between the FI scores and scores of HRQoL. Furthermore, it becomes plausible that good adherence to GFD functioned as a motivating factor of HRQoL. It could be imagined that adherence to GFD is the channel through which FI impacts are transmitted to HRQoL. With good adherence to GFD, the negative impacts of FI would be significantly reduced, and HRQoL would be improved. The results of the mediating effect of food security on good adherence to GFD and then good HRQoL are supported by a previous cross-sectional study conducted by [14]. The later study documented that FI influences adherence to a GFD and that most patients not adhering to a GFD were FI, while almost half of the patients adhering to a GFD were food secure. This result suggests that FI is highly dependent on adherence to a GFD. Furthermore, they documented that FI was negatively associated with HRQoL. The results of our study demonstrate a need for further empirical studies to identify factors enhancing the relationship between FI and HRQoL among patients with CD. Furthermore, it will be worth noting to find out the correlation between mediating factors and predictors of HRQoL to reach the maximum mediating effect and consequently enhance the overall relationship between FI and HRQoL among patients with CD.

## 5. Strength and Limitations

The main strength of this study is that it is the first study with a large sample size from Jordan to employ conceptual models to examine the moderation and mediation effects of adherence to GFD on the relationship between FI and HRQoL. This provides robust statistical analysis and objective insights into the association between these variables. The limitations of this study are the cross-sectional design and self-administered questionnaire, which is subjected to recall bias and restrict the generalizability of the results and limited by various factors, including gender, marital status, and monthly income, which could potentially restrict the applicability of results to broader populations. Additional confounding variables could face challenges in this type of study, including comorbidities, dietary habits, and psychosocial factors, which may influence FI and HRQoL among patients with CD.

## 6. Conclusions and Future Research

It could be concluded that there is a complicated interplay between FI and HRQoL in patients with CD that is impacted by several factors, including gender, marital status, and income.

Our findings show that FI is affected by following a GFD and has an impact on HRQoL. We found a clear, significant link between HRQoL and both FI and GFD adherence, indicating that reducing symptoms and complications associated with CD could lessen the impact of FI on HRQoL. It is worth noting that GFD adherence does not change the relationship between FI and HRQoL, suggesting that it could alleviate the negative effects of FI by improving CD management and nutritional status

Thus, interventions aimed at addressing FI among patients with CD should also consider strategies to support adherence to the GFD. Furthermore, the predominance of females among our study participants prompts consideration of how this demographic might influence the applicability of findings. While our results offer valuable insights into the association between CD and other variables, caution is advised when generalizing to populations with different demographic compositions. Future studies could broaden participant diversity. They should include a wider range of ages and socio-economic backgrounds. This would enhance the breadth. Such research would hold greater relevance. Conclusions drawn from this expanded participant pool would be more universally applicable. In summary, while FI can negatively affect the HRQoL of patients with CD, adherence to the GFD plays a crucial role in mitigating these effects. Future research and interventions should address FI and barriers to adherence to the GFD to optimize the health outcomes and well-being of individuals living with CD.

## Figures and Tables

**Table 1:** Characteristics of the study participants (*n* = 1162).

Variable	Categories	*N* (%)
Age *	28.52 ± 8.48	
**Gender**	Male	321 (27.6)
	Female	841 (72.4)
**Marital status**	Single without spouse	703 (60.5)
	Married	459 (39.5)
Monthly income (JOD) **	<500	672 (57.8)
	500–700	292 (25.1)
	>700	198 (17.0)
**Education level**	Secondary school or lower	635 (54.6)
	Undergraduate degree	479 (41.2)
	Postgraduate degree	48 (4.1)
Body Mass Index *	20.16 ± 2.3	
	Normal	270 (23.2)
	Overweight	832 (71.6)
	Obese	60 (5.2)

* Continuous variable, the data represented as mean ± SD. JOD—Jordanian Dinar. ** The average monthly wage in Jordan was JOD 535 in 2000 and increased to JOD 691 in 2021.

**Table 2:** Pearson correlation coefficient between the study variables (*n* = 1162).

Variable	FI	GFD Adherence
FI	-	0.489 **
GFD Adherence	0.489 **	-
HRQoL	−0.367 **	−0.353 **

** Correlation is significant at the 0.01 level (two-tailed); FI—Food Insecurity; GFD—Gluten-Free Diet; HRQoL—Health-Related Quality of Life.

**Table 3:** Multiple linear regression model for predicting HRQoL score.

Variable	Unstandardized Coefficients		Standardize Coefficients	*p*-Value	95.0% Confidence Interval for B	
	B	SE	Beta		Lower Bound	Upper Bound
**(Constant)**	42.336	3.089		0.000	36.275	48.397
Age	0.052	0.041	0.038	0.210	−0.029	0.133
BMI	0.134	0.142	0.026	0.346	−0.145	0.412
Gender (female)	−4.619	0.743	−0.178	≤0.001	−6.077	−3.162
Marital status (married)	−2.700	0.755	−0.114	≤0.001	−4.181	−1.220
Monthly income (JOD)						
500–700	2.370	0.788	0.088	0.003	0.824	3.916
>700	6.681	0.938	0.216	≤0.001	4.840	8.522
Education level						
Undergraduate	−0.520	0.740	−0.022	0.482	−1.971	0.931
Postgraduate	2.778	1.725	0.048	0.108	−0.607	6.163

BMI—Body Mass Index.

**Table 4:** Results from PROCESS macro testing GFD adherence mediation model.

**Effect ^a^, Variable**	**R^2^**	**F**	**β**	***p*-Value**
Direct effect of mediator (GFD adherence on HRQoL)	0.213	62.56	−0.638	≤0.0001
Direct effect of the predictor (FI) on mediator (GFD adherence)	0.246	94.15	0.288	≤0.0001
Total effect of predictor (FI) on HRQoL	0.178	28.97	−0.509	≤0.0001
Direct effect of predictor (FI) on HRQoL with inclusion of the mediator (GFD adherence t)	0.213	62.56	−0.406	≤0.0001
	**β**	**95% CL**		***p*-Value**
Indirect predictor (Food insecurity) on HRQoL	−0.184	−0.236	−0.134	≤0.0001

^a^ Adjusted for gender, marital status, and monthly income. FI—Food Insecurity; GFD—Gluten-Free Diet; HRQoL—Health-Related Quality of Life.

**Table 5:** Results from PROCESS macro testing GFD adherence moderating model.

^a^ Variable	R^2^	β	SE	t	*p*-Value	95.0% Confidence Interval	
						Lower Bound	Upper Bound
Direct effect of predictor (FI) on HRQoL	0.213	−0.399	0.173	−2.30	0.021	−0.739	−0.095
Direct effect of a moderator (GFD Adherence	0.213	−0.629	0.236	−2.66	0.007	−1.092	−0.166
Direct interactions effect (FI × GFD Adherence) on HRQoL	0.213	−0.0005	0.011	−0.043	0.965	−0.0224	0.021

^a^ Adjusted for gender, marital status, and monthly income. FI—Food Insecurity; GFD—Gluten-Free Diet; HRQoL—Health-Related Quality of Life.

## Data Availability

The data presented in this study are available on request from the corresponding author due to the research privacy and Ethical considerations.

## References

[1] Scherf K.A., Catassi C., Chirdo F., Ciclitira P.J., Feighery C., Gianfrani C., Koning F., Lundin K.E.A., Schuppan D., Smulders M.J.M. (2020). Recent Progress and Recommendations on Celiac Disease From the Working Group on Prolamin Analysis and Toxicity. Front. Nutr..

[2] Caio G., Volta U., Sapone A., Leffler D.A., De Giorgio R., Catassi C., Fasano A. (2019). Celiac disease: A comprehensive current review. BMC Med..

[3] King J.A., Jeong J., Underwood F.E., Quan J., Panaccione N., Windsor J.W., Coward S., Debruyn J., Ronksley P.E., Shaheen A.-A. (2020). Incidence of Celiac Disease Is Increasing Over Time: A Systematic Review and Meta-analysis. Am. J. Gastroenterol..

[4] Aljada B., Zohni A., El-Matary W. (2021). The Gluten-Free Diet for Celiac Disease and Beyond. Nutrients.

[5] Bascuñán K.A., Vespa M.C., Araya M. (2017). Celiac disease: Understanding the gluten-free diet. Eur. J. Nutr..

[6] Deepak C., Berry N., Vaiphei K., Dhaka N., Sinha S.K., Kochhar R. (2018). Quality of life in celiac disease and the effect of gluten-free diet. JGH Open.

[7] Al-Sunaid F.F., Al-Homidi M.M., Al-Qahtani R.M., Al-Ashwal R.A., Mudhish G.A., Hanbazaza M.A., Al-Zaben A.S. (2021). The influence of a gluten-free diet on health-related quality of life in individuals with celiac disease. BMC Gastroenterol..

[8] Muhammad H., Reeves S., Jeanes Y.M. (2019). Identifying and improving adherence to the gluten-free diet in people with coeliac disease. Proc. Nutr. Soc..

[9] Ma C., Singh S., Jairath V., Radulescu G., Ho S.K., Choi M.Y. (2022). Food Insecurity Negatively Impacts Gluten Avoidance and Nutritional Intake in Patients With Celiac Disease. J. Clin. Gastroenterol..

[10] Wieser H., Segura V., Ruiz-Carnicer Á., Sousa C., Comino I. (2021). Food Safety and Cross-Contamination of Gluten-Free Products: A Narrative Review. Nutrients.

[11] Siminiuc R., Ṭurcanu D. (2022). Food security of people with celiac disease in the Republic of Moldova through prism of public policies. Front. Public Health.

[12] Elsahoryi N., Al-Sayyed H., Odeh M., McGrattan A., Hammad F. (2020). Effect of COVID-19 on food security: A cross-sectional survey. Clin. Nutr. ESPEN.

[13] Pourhoseingholi M.A., Vahedi M., Rahimzadeh M. (2013). Sample size calculation in medical studies. Gastroenterol. Hepatol. Bed Bench.

[14] Marques B., Azevedo J., Rodrigues I., Rainho C., Gonçalves C. (2022). Food Insecurity Levels among University Students: A Cross-Sectional Study. Societies.

[15] Hosseini S.M., Soltanizadeh N., Mirmoghtadaee P., Banavand P., Mirmoghtadaie L., Shojaee-Aliabadi S. (2018). Gluten-free products in celiac disease: Nutritional and technological challenges and solutions. J. Res. Med. Sci..

[16] Baron R.M., Kenny D.A. (1986). The moderator–mediator variable distinction in social psychological research: Conceptual, strategic, and statistical considerations. J. Pers. Soc. Psychol..

[17] Violato M., Gray A. (2019). The impact of diagnosis on health-related quality of life in people with coeliac disease: A UK population-based longitudinal perspective. BMC Gastroenterol..

[18] Kasiulevičius V., Šapoka V., Filipavičiūtė R. (2006). Sample size calculation in epidemiological studies. Gerontologija.

[19] Patrick D., Erickson P. (1993). Health Policy, Quality of Life: Health Care Evaluation and Resource Allocation.

[20] Meysamie A., Taee F., Mohammadi-Vajari M.-A., Yoosefi-Khanghah S., Emamzadeh-Fard S., Abbassi M. (2014). Sample size calculation on web, can we rely on the results?. J. Med. Stat. Inform..

[21] McCrum-Gardner E. (2010). Sample size and power calculations made simple. Int. J. Ther. Rehabil..

[22] Haddad C., Sacre H., Obeid S., Salameh P., Hallit S. (2021). Validation of the Arabic version of the “12-item short-form health survey” (SF-12) in a sample of Lebanese adults. Arch. Public Health.

[23] Leffler D.A., Dennis M., Edwards George J.B., Jamma S., Magge S., Cook E.F., Schuppan D., Kelly C.P. (2009). A Simple Validated Gluten-Free Diet Adherence Survey for Adults with Celiac Disease. Clin. Gastroenterol. Hepatol..

[24] Naja F., Hwalla N., Fossian T., Zebian D., Nasreddine L. (2015). Validity and reliability of the Arabic version of the Household Food Insecurity Access Scale in rural Lebanon. Public Health Nutr..

[25] Degroot A.M.B., Dannenburg L., Vanhell J.G. (1994). Forward and Backward Word Translation by Bilinguals. J. Mem. Lang..

[26] Ware J.E., Kosinski M., Keller S.D. (1996). A 12-Item Short-Form Health Survey. Med. Care.

[27] Manjunath K., Christopher P., Gopichandran V., Rakesh P., George K., Prasad J.H. (2014). Quality of life of a patient with type 2 diabetes: A cross-sectional study in Rural South India. J. Fam. Med. Prim. Care.

[28] Hayes A.F. (2017). Introduction to Mediation, Moderation, and Conditional Process Analysis.

[29] Al Sarkhy A., El Mouzan M.I., Saeed E., Alanazi A., Alghamdi S., Anil S., Assiri A. (2015). Clinical Characteristics of Celiac Disease and Dietary Adherence to Gluten-Free Diet among Saudi Children. Pediatr. Gastroenterol. Hepatol. Nutr..

[30] Oyarzún A., Nakash T., Ayala J., Lucero Y., Araya M. (2016). Following Gluten Free Diet: Less Available, Higher Cost and Poor Nutritional Profile of Gluten-Free School Snacks. Int. J. Celiac Dis..

[31] Alimoradi Z., Kazemi F., Estaki T. (2015). Household food security in Iran: Systematic review of Iranian articles. Adv. Nurs. Midwifery.

[32] Khalifeh F., Riasatian M.S., Ekramzadeh M., Honar N., Jalali M. (2019). Assessing the Prevalence of Food Insecurity among Children with Celiac Disease: A Cross-sectional Study. J. Food Secur..

[33] Taghdir M., Honar N., Mazloomi S.M., Sepandi M., Ashourpour M., Salehi M. (2016). Dietary compliance in Iranian children and adolescents with celiac disease. J. Multidiscip. Healthc..

[34] Case S. (2005). The gluten-free diet: How to provide effective education and resources. Gastroenterology.

[35] Lee H.J., Anderson Z., Ryu D. (2014). Gluten Contamination in Foods Labeled as “Gluten Free” in the United States. J. Food Prot..

[36] Biagi F., Bianchi P.I., Marchese A., Trotta L., Vattiato C., Balduzzi D., Brusco G., Andrealli A., Cisarò F., Astegiano M. (2012). A score that verifies adherence to a gluten-free diet: A cross-sectional, multicentre validation in real clinical life. Br. J. Nutr..

[37] Biagi F., Andrealli A., Bianchi P.I., Marchese A., Klersy C., Corazza G.R. (2009). A gluten-free diet score to evaluate dietary compliance in patients with coeliac disease. Br. J. Nutr..

[38] Sdepanian V.L., Morais MB d.e., Fagundes-Neto U. (2001). Doença celíaca: Avaliação da obediência à dieta isenta de glúten e do conhecimento da doença pelos pacientes cadastrados na Associação dos Celíacos do Brasil (ACELBRA). Arq. Gastroenterol..

[39] Halmos E.P., Deng M., Knowles S.R., Sainsbury K., Mullan B., Tye-Din J.A. (2018). Food knowledge and psychological state predict adherence to a gluten-free diet in a survey of 5310 Australians and New Zealanders with coeliac disease. Aliment. Pharmacol. Ther..

[40] Hall N.J., Rubin G.P., Charnock A. (2013). Intentional and inadvertent non-adherence in adult coeliac disease. A cross-sectional survey. Appetite.

[41] Olsson C., Lyon P., Hörnell A., Ivarsson A., Sydner Y.M. (2009). Food That Makes You Different: The Stigma Experienced by Adolescents with Celiac Disease. Qual. Health Res..

[42] Casella S., Zanini B., Lanzarotto F., Villanacci V., Ricci C., Lanzini A. (2012). Celiac Disease in Elderly Adults: Clinical, Serological, and Histological Characteristics and the Effect of a Gluten-Free Diet. J. Am. Geriatr. Soc..

[43] Vilppula A., Kaukinen K., Luostarinen L., Krekelä I., Patrikainen H., Valve R., Luostarinen M., Laurila K., Mäki M., Collin P. (2011). Clinical benefit of gluten-free diet in screen-detected older celiac disease patients. BMC Gastroenterol..

[44] Rajpoot P., Sharma A., Harikrishnan S., Baruah B.J., Ahuja V., Makharia G.K. (2015). Adherence to gluten-free diet and barriers to adherence in patients with celiac disease. Indian J. Gastroenterol..

[45] Hughey J.J., Ray B.K., Lee A.R., Voorhees K.N., Kelly C.P., Schuppan D. (2017). Self-reported dietary adherence, disease-specific symptoms, and quality of life are associated with healthcare provider follow-up in celiac disease. BMC Gastroenterol..

[46] van Hees N.J.M., Van der Does W., Giltay E.J. (2013). Coeliac disease, diet adherence and depressive symptoms. J. Psychosom. Res..

[47] Sainsbury K., Marques M.M. (2018). The relationship between gluten free diet adherence and depressive symptoms in adults with coeliac disease: A systematic review with meta-analysis. Appetite.

[48] Pietzak M.M. (2005). Follow-up of patients with celiac disease: Achieving compliance with treatment. Gastroenterology.

[49] Jeanes Y. (2018). Cost, availability and nutritional composition comparison between gluten free and gluten containing food staples provided by food outlets and internet food delivery services between two areas of London with differing UK deprivation indices. Coeliac UK Delegates Brochure.

[50] Singh J., Whelan K. (2011). Limited availability and higher cost of gluten-free foods. J. Hum. Nutr. Diet..

[51] Barratt S.M., Leeds J.S., Sanders D.S. (2011). Quality of life in Coeliac Disease is determined by perceived degree of difficulty adhering to a gluten-free diet, not the level of dietary adherence ultimately achieved. J. Gastrointestin. Liver Dis..

[52] Zarkadas M., Cranney A., Case S., Molloy M., Switzer C., Graham I.D., Butzner J.D., Rashid M., Warren R.E., Burrows V. (2006). The impact of a gluten-free diet on adults with coeliac disease: Results of a national survey. J. Hum. Nutr. Diet..

[53] Aljahdali A.A., Na M., Leung C.W. (2024). Food insecurity and health-related quality of life among a nationally representative sample of older adults: Cross-sectional analysis. BMC Geriatr..

[54] Casellas F., Rodrigo L., Vivancos J.L., Riestra S., Pantiga C., Baudet J., Junquera F., Diví V.P., Abadia C., Papo M. (2008). Factors that impact health-related quality of life in adults with celiac disease: A multicenter study. World J. Gastroenterol..

[55] Rustagi S., Choudhary S., Khan S., Jain T. (2020). Consequential Effect of Gluten-Free Diet on Health-Related Quality of Life in Celiac Populace-A Meta-Analysis. Curr. Res. Nutr. Food Sci. J..

[56] Jones A.D. (2017). Food Insecurity and Mental Health Status: A Global Analysis of 149 Countries. Am. J. Prev. Med..

[57] Ali S.H., Alqurneh R., Abu Sneineh A., Ghazal B., Agraib L., Abbasi L., Mazzawi T., Rifaei S.M. (2023). The Prevalence of Anxiety and Depressive Symptoms among Patients with Celiac Disease in Jordan. Cureus.

[58] Zysk W., Głąbska D., Guzek D. (2018). Social and Emotional Fears and Worries Influencing the Quality of Life of Female Celiac Disease Patients Following a Gluten-Free Diet. Nutrients.

[59] Alahmari T.M., Asiri A.J., Bilali R.M., Qahtani A.S., Alqarni M.A., Almalwi F.A. (2018). Quality of Life and Wellbeing of Patients with Celiac Disease in Aseer Region of Saudi Arabia. Int. J. Med. Res. Prof..

[60] Pourhoseingholi M.A., Rostami-Nejad M., Barzegar F., Rostami K., Volta U., Sadeghi A., Honarkar Z., Salehi N., Asadzadeh-Aghdaei H., Baghestani A.R. (2017). Economic burden made celiac disease an expensive and challenging condition for Iranian patients. Gastroenterol. Hepatol. Bed Bench.

